# Systemic immune-inflammation index during treatment predicts prognosis and guides clinical treatment in patients with nasopharyngeal carcinoma

**DOI:** 10.1007/s00432-022-04506-z

**Published:** 2023-01-03

**Authors:** Xiaofei Yuan, Huiru Feng, Haoran Huang, Junzheng Li, Shuting Wu, Yue Yuan, Linchong Cui, Danfan Lin, Zilu Chen, Wenxuan Lu, Xiong Liu, Xiaohong Peng, Fan Wang

**Affiliations:** 1 Department of Otolaryngology-Head and Neck Surgery, Nanfang Hospital, Southern Medical University, Jingxi Street, Baiyun District, Guangzhou, 510515 People’s Republic of China; 2 Department of Otolaryngology-Head and Neck Surgery, Zhujiang Hospital, Southern Medical University, Guangzhou, 510515 People’s Republic of China

**Keywords:** Nasopharyngeal carcinoma, Systemic immune-inflammation index, Uncontrolled, Prognosis, During treatment

## Abstract

**Purpose:**

Systemic immune-inflammation index (SII) has been demonstrated to be closely associated with the poor prognosis of nasopharyngeal carcinoma (NPC). However, the role of SII during treatment of NPC has not been reported. This study aimed to determine the prognostic value of SII during treatment for NPC patients.

**Methods:**

A total of 759 patients diagnosed with NPC were included in this retrospective study (393 in training cohort and 366 in validation cohort). The correlation between variables was analyzed by the chi-squared test, the Fisher’s exact test or the likelihood test. Kaplan–Meier method and log-rank test were used to analyze progression-free survival (PFS) and overall survival (OS). The independent prognostic factors were determined by multivariate analysis of Cox proportional hazards regression model. The uncontrolled risk was analyzed by Logistic regression. Receiver operating characteristic (ROC) curves were used to assess prognostic value.

**Results:**

The optimal cut-off point for the SII during treatment was 937.32. High SII during treatment group had higher uncontrolled risk than low SII during treatment group (*p* = 0.008). In multivariate Cox proportional hazard models analysis, SII during treatment was an independent prognostic factor for 5-year PFS (*p* < 0.001) and 5-year OS (*p* < 0.001). All results were found in the training cohort and confirmed in the validation cohort.

**Conclusions:**

The SII during treatment is a promising indicator of predicting the survival in NPC patients, especially the risk of uncontrolled occurrence. By monitoring the SII during treatment, it is possible to better evaluate the treatment effect and formulate personalized treatment.

**Supplementary Information:**

The online version contains supplementary material available at 10.1007/s00432-022-04506-z.

## Introduction

Nasopharyngeal carcinoma (NPC) is a malignant tumor of the head and neck originating from the nasopharyngeal epithelium, it is closely related to Epstein–Barr virus (EBV) infection, and has apparent regional and epidemic characteristics (Chen et al. 2019; Sung et al. 2021). Since it is sensitive to chemo-radiation, radiotherapy with or without chemotherapy is the main treatment for NPC patients. However, the survival of one-third of patients remains poor because of local recurrence and distant metastasis (Wu et al. 2014; Lee et al. 2015). In addition, some patients have residual disease or develop recurrent disease at the primary or regional site (Mao et al. 2016). Therefore, finding an effective and accurate prognostic indicator is crucial to improve the clinical management of NPC.

In recent years, increasing evidence has shown that activation of inflammation is a crucial mechanism for the recurrence and metastasis of cancer (Balkwill and Mantovani 2010). Inflammatory parameters such as the neutrophil to lymphocyte ratio (NLR) (Chua et al. 2016), platelet-to-lymphocyte ratio (PLR) (Fang et al. 2020), systemic immune-inflammation index (SII) (Hu et al. 2014) and systemic inflammation response index (SIRI) (Valero et al. 2019) have been identified as prognostic biomarkers in multiple cancers, including gastric cancer, hepatocellular cancer, oral cavity cancer and NPC. A previous study was followed by Zeng et al. (2020), which conducted a retrospective study including 559 NPC patients and 500 chronic rhinitis patients. This study reported that pre-treatment inflammatory parameters (including NLR, PLR, SII and SIRI) in patients with NPC were significantly higher than those in patients with chronic rhinitis, and could be used as prognostic indicators in NPC patients. However, since most of the relevant studies mainly focused on prognosis with pre-treatment inflammatory parameters, to our knowledge, no data on the investigation of the prognostic potential of the inflammatory parameters during treatment for NPC are present. In addition, some patients have residual tumor or metastasis within 6 months after the end of the treatment, these patients were called uncontrolled in clinical practice. Radio-resistance is an important cause of uncontrolled occurrence in NPC, and studies reported that radio-resistant tumor cells could cause an increase in inflammation during treatment (Nantajit et al. 2015; Yuan et al. 2021). Therefore, observing changes in inflammatory parameters during treatment was of great significance in predicting the degree of uncontrolled risk.

Therefore, a total 759 NPC patients were included in this retrospective study. The objective of this study was to evaluate the prognostic of inflammatory parameters during treatment. In addition, whether the inflammatory parameters during treatment provides a credible prognostic evaluation for uncontrolled patients was also assessed. This study aimed to determine the prognostic value of inflammatory parameters for NPC patients during treatment, and determine whether inflammatory parameters can enhance the survival prediction, and promote development of individualized treatment approach for NPC.

## Materials and methods

### Case selection

We retrospectively recruited 759 patients who were diagnosed with NPC at NanFang Hospital of Southern Medical University from December 2007 to December 2015. All patients were treated according to the guidelines (The treatment method was detailed in Supplementary file 1). All patients were divided into the training cohort and validation cohort by random number. The eighth edition of the American Joint Committee on Cancer (AJCC) staging system was used for stage classification. This study was approved by the Ethics Committee of Nanfang Hospital of Southern Medical University (Ethical review approval no.: NFEC-2017-165).

### Inclusion and exclusion criteria

The inclusion criteria in this study comprised of: (a) patients with histopathological confirmation of NPC; (b) patients with complete medical records and treatment time records; and (c) patients with at least one complete record of haematological indicators during treatment. The exclusion criteria were as follows: (a) patients with non-WHO pathological types; (b) patients with prior malignancy; and (c) non-first-treatment; (d) patients with uncontrolled infection.

### Haematological examination

The peripheral blood of all patients was collected and tested for neutrophil, lymphocyte, platelet and monocyte counts within 2 days before the start of each chemotherapy and 3 days after the end of each chemotherapy, or at least once a week during the entire treatment period. The measurements of plasma EBV DNA were performed within 1 month before therapy. If the patient had II bone marrow suppression, peripheral blood cells were monitored twice a week, while if the patient had III or worse bone marrow suppression, short-acting recombinant human granulocyte colony stimulating factor therapy was given. The peripheral blood data with the lowest leukocyte count was selected during treatment. All peripheral blood cell and EBV DNA assessments were performed in our hospital’s institutional laboratory according to standard operating procedures (The detection method for EBV DNA in Supplementary file 2). The cut-off level chosen to classify the patients into the negative and positive EBV DNA groups was 500 copies/mL before treatment, referring to the threshold of Laboratory Medicine Center, Nanfang Hospital, Southern Medical University. The NLR was defined as neutrophil/lymphocyte; the PLR was defined as platelet/lymphocyte; the SII was defined as (neutrophil*platelet)/lymphocyte; the SIRI was defined as (neutrophil*monocyte)/lymphocyte.

### Follow-up

Patients were followed up at 3, 6, and 12 months in the first year after treatment, every 6 months in the second and third years, and once a year thereafter. Follow-up assessments included head and neck physical examination, nasopharyngeal endoscopy, chest radiograph, abdominal ultrasound, peripheral blood examination, nasopharyngeal and neck magnetic resonance imaging (MRI) and emission computed tomography (ECT) or the whole-body positron emission tomography-computed tomography (PET-CT). For cases of suspected nasopharyngeal and neck tumor recurrence or distant metastasis to cervical lymph nodes, biopsy or acupuncture biopsy of the suspected site was performed to confirm the diagnosis. Progression-free survival (PFS) was defined as the time from the initial pathological diagnosis of NPC to the date of disease progression or death from any cause. Overall survival (OS) was defined as the time between the initial pathological diagnosis of NPC and all-cause death, or at the last follow-up. Uncontrolled was defined as the tumor remained or metastases appeared within 6 months after the end of treatment.

### Statistical analysis

The Statistical Packages for Social Science version 23.0 (IBM, Corporation) was used. The optimal cut-off value of NLR, PLR, SII and SIRI were determined using X-tile 3.6.1 software (Robert L Camp, Yale University, New Haven, CT, USA) (Camp et al. 2004; Lolli et al. 2016). The cut-off value was plotted by X-tile 3.6.1 software, and other figures were plotted by GraphPad Prism V8.0. The chi-squared test, Fisher’s exact test or likelihood test was used to explore the baseline balance between the inflammatory parameters groups. Survival curves were analyzed using the Kaplan–Meier method and compared using the log-rank test. Univariate and multivariate Cox proportional hazards regressions were conducted to evaluate the prognostic significance of each variable with respect to PFS and OS. Logistic regression was used to estimate odds ratio (OR) and 95% CI in order to evaluate the association between clinical characteristics and uncontrolled rate. Receiver operating characteristic (ROC) curve analysis was performed to compare the different prognostic values. All results were validated by the validation cohort. A two-tailed *p* value of less than 0.05 was considered as statistically significant.

## Results

### The optimal cut-off values of inflammatory parameters

**Fig. 1:**
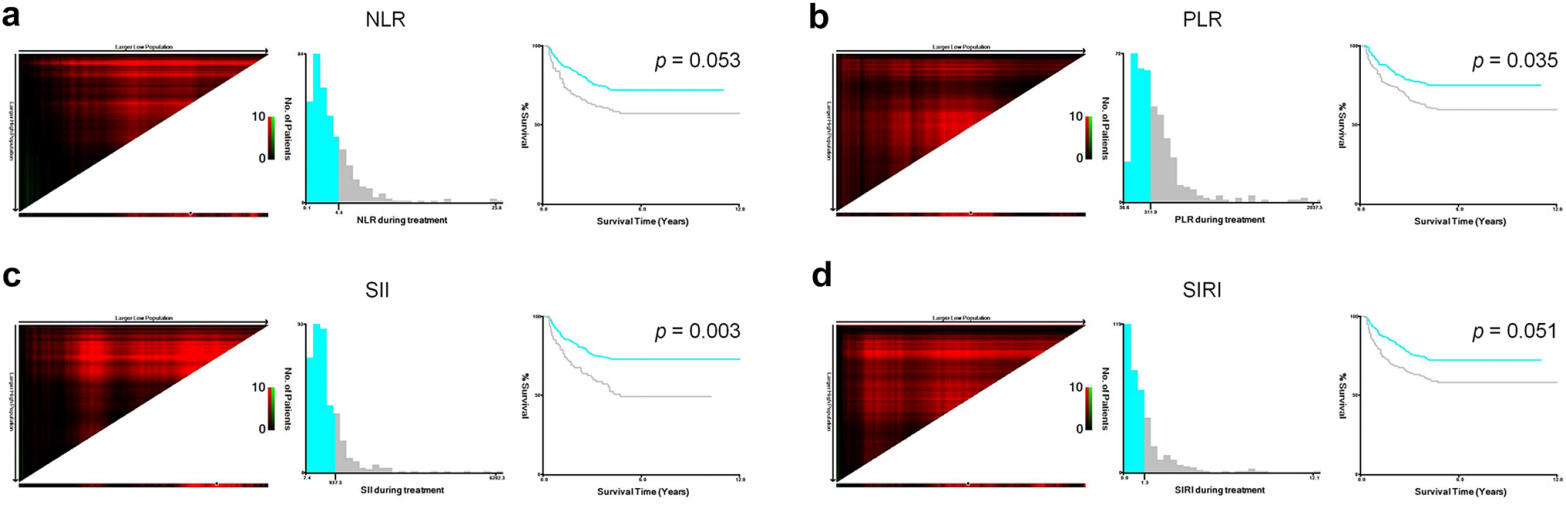
X-tile analysis of survival data of NPC patients. **a** The optimal cut-off value for the NLR was 4.38 (chi square = 9.184, *p* = 0.053); **b** The optimal cut-off value for the PLR was 311.86 (chi square = 10.112, *p* = 0.035); **c** The optimal cut-off value for the SII was 937.32 (chi square = 15.732, *p* = 0.003); **d** The optimal cut-off value for the SIRI was 1.27 (chi square = 9.291, *p* = 0.051)

The X-tile 3.6.1 software was used to evaluate the optimal cutoff value of the NLR, PLR, SII and SIRI for progression outcome in training cohort. The cut-off values of the NLR, PLR, SII and SIRI were 4.38 (*p* = 0.053), 311.86 (*p* = 0.035), 937.32 (*p* = 0.003) and 1.27 (*p* = 0.051), respectively (Fig. 1). Since only the cut-off values of PLR and SII were statistically significant after being calculated by X-tile software, PLR and SII were included in the subsequent statistical analysis.

### Baseline patient characteristics

A total of 393 and 366 patients were included in the training cohort and validation cohort, respectively. The baseline characteristics of the patients are shown in Tables 1 and 2. In the training cohort, there were 288 males (73.3%) and 105 females (26.7%). The median follow-up time was 61 months. During the long-term follow-up, 123 patients (31.3%) experienced disease progression, and 63 patients (16.0%) died. In the validation cohort, there were 258 males (70.5%) and 108 females (29.5%). The median follow-up time was 59 months. During the long-term follow-up, 110 patients (30.1%) experienced disease progression, and 60 patients (16.4%) died. Table 1 shows the correlations between SII during treatment and patient clinical characteristics in the two cohorts. In training cohort, SII during treatment was significantly correlated with age, tumor stage and metastasis (*p* < 0.05). However, there was no significant correlation in validation cohort. Table 2 shows the correlations between PLR during treatment and patient clinical characteristics in the two cohorts. In training cohort, PLR during treatment was significantly correlated with EBV DNA status, tumor stage and metastasis (*p* < 0.05). In validation cohort, there was no correlation between PLR during treatment and EBV DNA status, tumor stage and metastasis. In addition, the baseline characteristics for the training cohort and validation cohort are shown in Supplementary Table 1. The results showed that there was no significant difference in baseline characteristics between the two cohorts.

**Table 1:** Baseline characteristics in training and validation cohorts according to SII

Variables	Training cohort (*n* = 393)			Validation cohort (*n* = 366)		
	SII		*p*	SII		*p*
	≤ 937.32 No. (%)	> 937.32 No. (%)		≤ 937.32 No. (%)	> 937.32 No. (%)	
Gender			0.191			0.972
Male	224 (71.8)	64 (79.0)		209 (70.4)	49 (71.0)	
Female	88 (28.2)	17 (21.0)		88 (29.6)	20 (29.0)	
Age			0.010^*^			0.454
≤ 55	250 (80.1)	54 (66.7)		236 (79.5)	52 (75.4)	
> 55	62 (19.9)	27 (33.3)		61 (20.5)	17 (24.6)	
EBV DNA status			0.963			0.642
Negative	128 (41.0)	33 (40.7)		134 (45.1)	29 (42.0)	
Positive	184 (59.0)	48 (59.3)		163 (54.9)	40 (58.0)	
AJCC stage (8th)			0.205			0.189
I–II	54 (17.3)	19 (23.5)		64 (21.5)	10 (14.5)	
III–IVb	258 (82.7)	62 (76.5)		233 (78.5)	59 (85.5)	
Tumor stage			0.015^*^			0.677
T1–T2	112 (35.9)	41 (50.6)		120 (40.4)	26 (37.7)	
T3–T4	200 (64.1)	40 (49.4)		177 (59.6)	43 (62.3)	
Node stage			0.729			0.166
N0–N1	132 (42.3)	36 (44.4)		126 (42.4)	23 (33.3)	
N2–N3	180 (57.7)	45 (55.6)		171 (57.6)	46 (66.7)	
Metastasis			0.019^*^			0.254
Non-metastasis	303 (97.1)	74 (91.4)		285 (96.0)	64 (92.8)	
Metastasis	9 (2.9)	7 (8.6)		12 (4.0)	5 (7.2)	
WHO pathologic type			0.616			0.532
TypeI	1 (0.3)	0 (0)		3 (1.0)	0 (0)	
TypeII	22 (7.1)	4 (4.9)		26 (8.8)	6 (8.7)	
TypeIII	289 (92.6)	77 (95.1)		268 (90.2)	63 (91.3)	

*p < 0.05

*SII* Systemic immune-inflammation index, *EBV DNA* Epstein-Barr virus DNA, *AJCC* American Joint Committee on Cancer, *WHO* World Health Organization

**Table 2:** Baseline characteristics in training and validation cohorts according to PLR

Characteristic	Training cohort (*n* = 393)			Validation cohort (*n* = 366)		
	PLR		*p*	PLR		*p*
	≤ 311.86 No. (%)	> 311.86 No. (%)		≤ 311.86 No. (%)	> 311.86 No. (%)	
Gender			0.176			0.001^*^
Male	162 (76.1)	126 (70.0)		149 (78.0)	109 (62.3)	
Female	51 (23.9)	54 (30.0)		42 (22.0)	66 (37.7)	
Age			0.305			0.857
≤ 55	169 (79.3)	135 (75.0)		151 (79.1)	137 (78.3)	
> 55	44 (20.7)	45 (25.0)		40 (20.9)	38 (21.7)	
EBV DNA status			0.016^*^			0.211
Negative	99 (46.5)	62 (34.4)		91 (47.6)	72 (41.1)	
Positive	114 (53.5)	118 (65.6)		100 (52.4)	103 (58.9)	
AJCC stage (8th)			0.053			0.015^*^
I–II	47 (22.1)	26 (14.4)		48 (25.1)	26 (14.9)	
III–IVb	166 (77.9)	154 (85.6)		143 (74.9)	149 (85.1)	
Tumor stage			0.036^*^			0.146
T1–T2	93 (43.7)	60 (33.3)		83 (43.5)	63 (36.0)	
T3–T4	120 (56.3)	120 (66.7)		108 (56.5)	112 (64.0)	
Node stage			0.224			0.049^*^
N0–N1	97 (45.5)	71 (39.4)		87 (45.5)	62 (35.4)	
N2–N3	116 (54.5)	109 (60.6)		104 (54.5)	113 (64.6)	
Metastasis			< 0.001^*^			0.290
Non-metastasis	221 (99.1)	166 (92.2)		180 (94.2)	169 (96.6)	
Metastasis	2 (0.9)	14 (7.8)		11 (5.8)	6 (3.4)	
WHO pathologic type			0.493			0.727
TypeI	1 (0.5)	0 (0)		2 (1.0)	1 (0.6)	
TypeII	13 (6.1)	13 (7.2)		15 (7.9)	17 (9.7)	
TypeIII	199 (93.4)	167 (92.8)		174 (91.1)	157 (89.7)	

*p < 0.05

*PLR* Platelet-lymphocyte ratio, *EBV DNA* Epstein-Barr virus DNA, *AJCC* American Joint Committee on Cancer, *WHO* World Health Organization

### Kaplan–Meier method and log-rank test

**Fig. 2:**
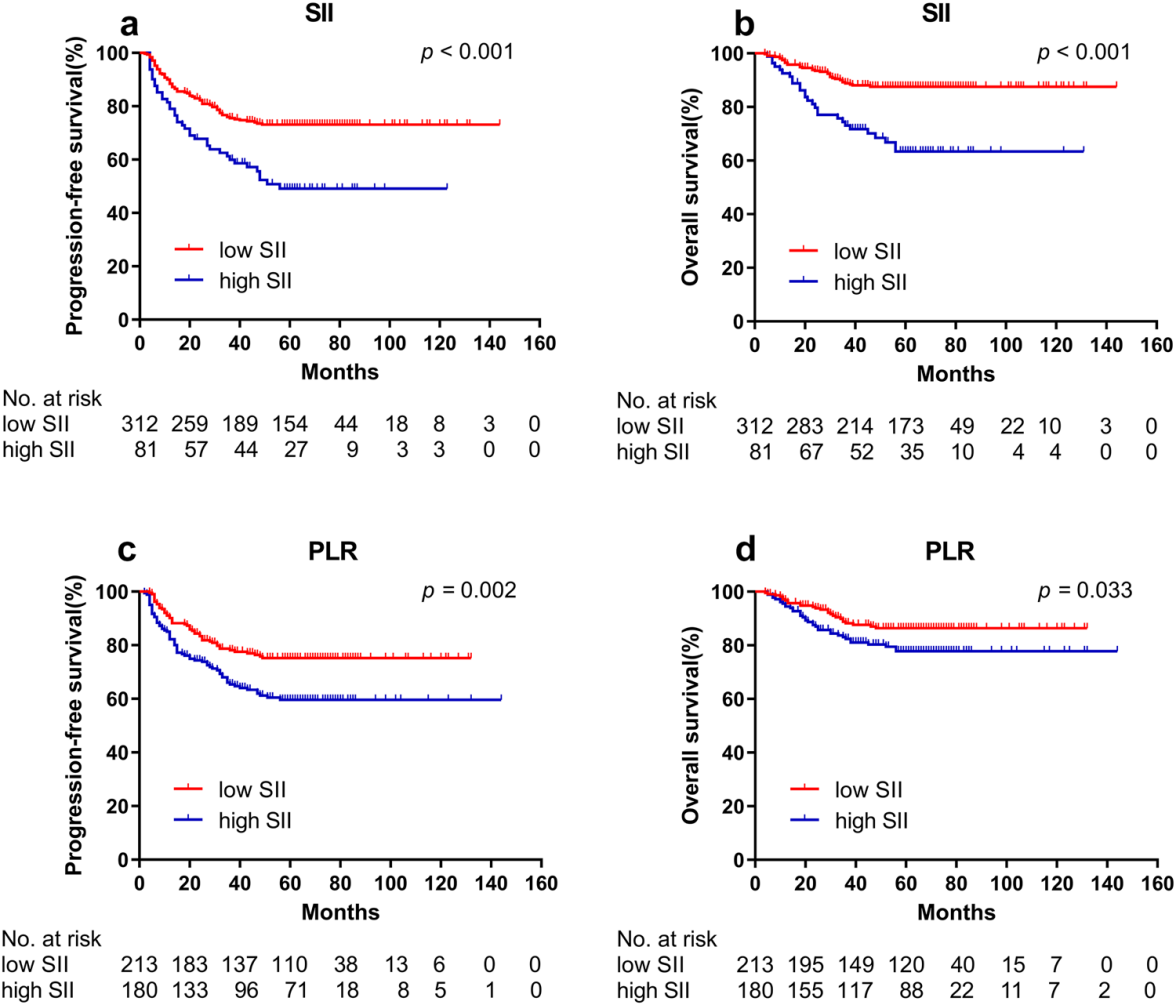
Kaplan–Meier curves for PFS and OS between different groups in training cohort. **a** Low SII and high SII group for PFS; **b** Low SII and high SII group for OS; **c** Low PLR and high PLR group for PFS; **d** Low PLR and high PLR group for OS

**Fig. 3:**
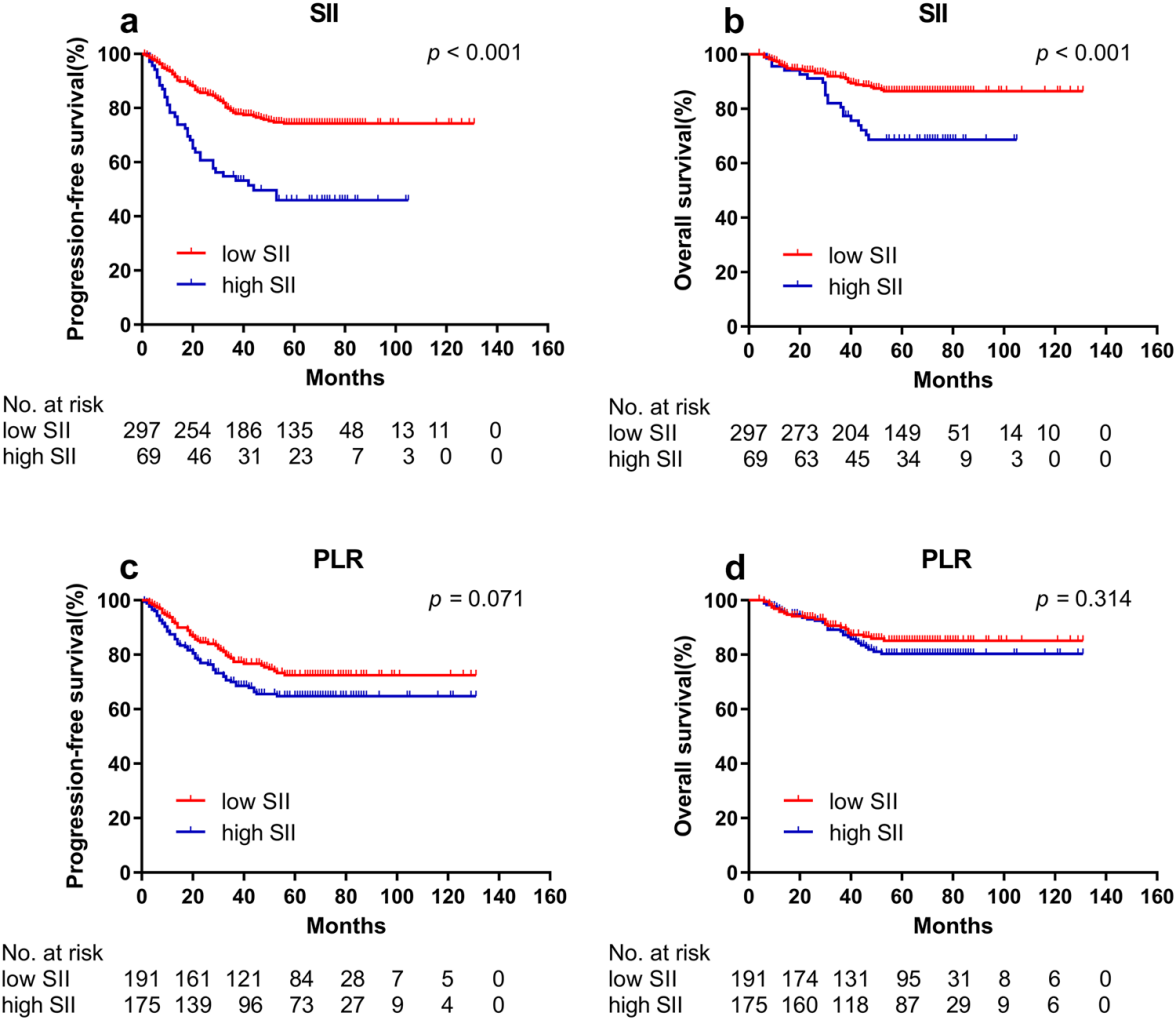
Kaplan–Meier curves for PFS and OS between different groups in validation cohort. **a** Low SII and high SII group for PFS; **b** Low SII and high SII group for OS; **c** Low PLR and high PLR group for PFS; **d** Low PLR and high PLR group for OS

Kaplan–Meier survival curves based on SII during treatment and PLR during treatment for survival analysis of training and validation cohorts are shown in Figs. 2 and 3. In the training cohort, the higher SII group demonstrated poorer PFS (*p* < 0.001) and OS (*p* < 0.001) compared to the lower SII group. And the higher PLR group demonstrated poorer PFS (*p* = 0.002) and OS (*p* = 0.033) compared to the lower PLR group. The survival results of SII during treatment were confirmed in the validation cohort (all *p* < 0.001). However, in the validation cohort, the higher PLR group were not significant associated to poorer PFS (*p* = 0.071) and OS (*p* = 0.314). Therefore, only SII during treatment was included in subsequent univariate and multivariate analyses.

### Univariate and multivariate Cox regression analysis

In the univariate Cox regression model, age (*p* = 0.016), AJCC stage (*p* < 0.001), node stage (*p* = 0.003), metastasis (*p* = 0.024), EBV DNA (*p* = 0.001) and SII during treatment (*p* < 0.001) were significantly associated with PFS in training cohort. Age (*p* = 0.007), AJCC stage (*p* = 0.030), tumor stage (*p* = 0.025), metastasis (*p* = 0.017), EBV DNA (*p* = 0.007) and SII during treatment (*p* < 0.001) were significantly associated with PFS in validation cohort. Age (*p* < 0.001), AJCC stage (*p* < 0.001), node stage (*p* = 0.032), metastasis (*p* = 0.010) and SII during treatment (*p* < 0.001) were significantly associated with OS in training cohort. Age (*p* < 0.001), AJCC stage (*p* = 0.026), tumor stage (*p* = 0.016), metastasis (*p* < 0.001), EBV DNA (*p* = 0.003) and SII during treatment (*p* = 0.001) were significantly associated with OS in validation cohort (Table 3). All variables reaching statistical significance in univariate analysis were included in multivariate Cox regression analysis.

**Table 3:** Univariate Cox regression analysis of PFS and OS in training and validation cohorts

Variables	Training cohort (*n* = 393)		Validation cohort (*n* = 366)	
	HR (95% CI)	*p*	HR (95% CI)	*p*
PFS				
Gender (female vs. male)	0.733 (0.471–1.140)	0.168	0.767 (0.495–1.187)	0.234
Age (> 55 vs. ≤ 55)	1.628 (1.096–2.418)	0.016^*^	1.771 (1.165–2.692)	0.007^*^
AJCC stage (8th) (III–IVb vs. I–II)	3.230 (1.636–6.376)	< 0.001^*^	1.862 (1.061–3.268)	0.030^*^
Tumor stage (T_3_–_4_ vs. T_1_–_2_)	1.285 (0.880–1.878)	0.195	1.598 (1.059–2.411)	0.025^*^
Node stage (N_2_–_3_ vs. N_0_–_1_)	1.817 (1.231–2.682)	0.003^*^	1.154 (0.780–1.706)	0.474
Metastasis (Yes vs. No)	2.282 (1.113–4.680)	0.024^*^	2.303 (1.163–4.562)	0.017^*^
WHO pathologic type (TypeIII vs. TypeII vs. TypeI)	0.719 (0.391–1.322)	0.288	1.140 (0.628–2.070)	0.667
EBV DNA (positive vs. negative)	1.942 (1.299–2.903)	0.001^*^	1.737 (1.162–2.597)	0.007^*^
SII during treatment (> 937.32 vs. ≤ 937.32)	2.216 (1.449–3.121)	< 0.001^*^	2.670 (1.785–3.993)	< 0.001^*^
OS				
Gender (female vs. male)	0.680 (0.362–1.278)	0.231	0.779 (0.425–1.429)	0.420
Age (> 55 vs. ≤ 55)	2.615 (1.573–4.347)	< 0.001^*^	2.797 (1.630–4.799)	< 0.001^*^
AJCC stage (8th) (III–IVb vs. I–II)	15.649 (2.169–112.90)	< 0.001^*^	2.849 (1.136–7.146)	0.026^*^
Tumor stage (T_3_–_4_ vs. T_1_–_2_)	1.314 (0.777–2.225)	0.308	2.111 (1.150–3.873)	0.016^*^
Node stage (N_2_–_3_ vs. N_0_–_1_)	1.807 (1.053–3.102)	0.032^*^	1.159 (0.673–1.997)	0.595
Metastasis (Yes vs. No)	3.044 (1.311–7.069)	0.010^*^	4.702 (2.220–9.959)	< 0.001^*^
WHO pathologic type (TypeIII vs. TypeII vs. TypeI)	1.137 (0.415–3.113)	0.803	1.457 (0.564–3.767)	0.437
EBV DNA (positive vs. negative)	1.554 (0.912–2.647)	0.105	2.500 (1.363–4.586)	0.003^*^
SII during treatment (> 937.32 vs. ≤ 937.32)	3.204 (1.939–5.295)	< 0.001^*^	2.494 (1.439–4.320)	0.001^*^

*p < 0.05

*AJCC* American Joint Committee on Cancer, *WHO* World Health Organization, *EBV DNA* Epstein-Barr virus DNA, *SII* Systemic immune-inflammation index

In the multivariate Cox regression model, the EBV DNA and SII during treatment were still independent risk factors in NPC patients for PFS in the training cohort and validation cohort (all *p* < 0.05). The age and SII during treatment were still found to be independent risk factors in NPC patients for OS in the training cohort and validation cohort (all *p* < 0.05) (Table 4).

**Table 4:** Multivariate Cox regression analysis of PFS and OS in training and validation cohorts

Variables	Training cohort (*n* = 393)		Validation cohort (*n* = 366)	
	HR (95% CI)	*p*	HR (95% CI)	*p*
PFS				
Age (> 55 vs. ≤ 55)	1.542 (1.027–2.314)	0.037^*^	1.424 (0.918–2.210)	0.115
AJCC stage (8th) (III–IVb vs. I–II)	2.576 (1.208–5.494)	0.014^*^	1.280 (0.635–2.581)	0.490
Tumor stage (T_3_–_4_ vs. T_1_–_2_)	–	–	1.242 (0.742–2.080)	0.409
Node stage (N_2_–_3_ vs. N_0_–_1_)	1.259 (0.809–1.959)	0.307	–	–
Metastasis (Yes vs. No)	1.569 (0.753–3.269)	0.229	1.394 (0.682–2.849)	0.363
EBV DNA (positive vs. negative)	1.669 (1.110–2.509)	0.014^*^	1.551 (1.026–2.344)	0.037^*^
SII during treatment (> 937.32 vs. ≤ 937.32)	2.117 (1.432–3.129)	< 0.001^*^	2.483 (1.647–3.744)	< 0.001^*^
OS				
Age (> 55 vs. ≤ 55)	2.361 (1.397–3.991)	0.001^*^	2.135 (1.209–3.772)	0.009^*^
AJCC stage (8th) (III–IVb vs. I–II)	16.534 (2.203–124.09)	0.006^*^	1.687 (0.555–5.132)	0.357
Tumor stage (T_3_–_4_ vs. T_1_–_2_)	–	–	1.258 (0.599–2.642)	0.544
Node stage (N_2_–_3_ vs. N_0_–_1_)	1.094 (0.622–1.921)	0.756	–	–
Metastasis (Yes vs. No)	1.706 (0.719–4.050)	0.226	2.651 (1.205–5.831)	0.015^*^
EBV DNA (positive vs. negative)	–	–	1.917 (1.031–3.566)	0.040^*^
SII during treatment (> 937.32 vs. ≤ 937.32)	2.975 (1.772–4.996)	< 0.001^*^	2.100 (1.196–3.688)	0.010^*^

*p < 0.05

### Relationship between uncontrolled rate and clinical characteristics

We further assessed which clinical characteristics were risk factors for uncontrolled occurrence. In the univariate logistic regression analysis, the AJCC stage (*p* = 0.046), metastasis (*p* = 0.040), EBV DNA (*p* = 0.035) and SII during treatment (*p* = 0.008) were risk factors for uncontrolled in training cohort. The age (*p* = 0.002) and SII during treatment (*p* = 0.003) were risk factors for uncontrolled in validation cohort (Table 5).

**Table 5:** Univariate logistic regression analysis of different characteristics as a function of uncontrolled rate (Uncontrolled, Non-uncontrolled) in training and validation cohorts

Variables	Training cohort (*n* = 393)		Validation cohort (*n* = 366)	
	OR (95% CI)	*p*	OR (95% CI)	*p*
Gender (female vs. male)	1.018 (0.475–2.181)	0.964	0.548 (0.201–1.493)	0.239
Age (> 55 vs. ≤ 55)	1.299 (0.603–2.798)	0.505	3.558 (1.573–8.052)	0.002^*^
AJCC stage (8th) (III-IVb vs. I-II)	4.360 (1.024–18.557)	0.046^*^	2.024 (0.591–6.391)	0.262
Tumor stage (T_3_–_4_ vs. T_1_–_2_)	1.197 (0.590–2.428)	0.619	1.877 (0.768–4.585)	0.167
Node stage (N_2_–_3_ vs. N_0_–_1_)	1.865 (0.894–3.890)	0.097	1.106 (0.488–2.510)	0.809
Metastasis (Yes vs. No)	3.475 (1.061–11.383)	0.040^*^	0.810 (0.103–6.361)	0.841
WHO pathologic type (TypeIII vs. TypeII vs. TypeI)	0.475 (0.178–1.267)	0.137	1.356 (0.337–5.456)	0.668
EBV DNA (positive vs. negative)	2.318 (1.063–5.056)	0.035^*^	2.301 (0.943–5.618)	0.067
SII during treatment (> 937.32 vs. ≤ 937.32)	2.626 (1.284–5.370)	0.008^*^	3.366 (1.558–8.159)	0.003^*^

*p < 0.05

*AJCC* American Joint Committee on Cancer, *WHO* World Health Organization, *EBV DNA* Epstein-Barr virus DNA, *SII* Systemic immune-inflammation index

**Fig. 4:**
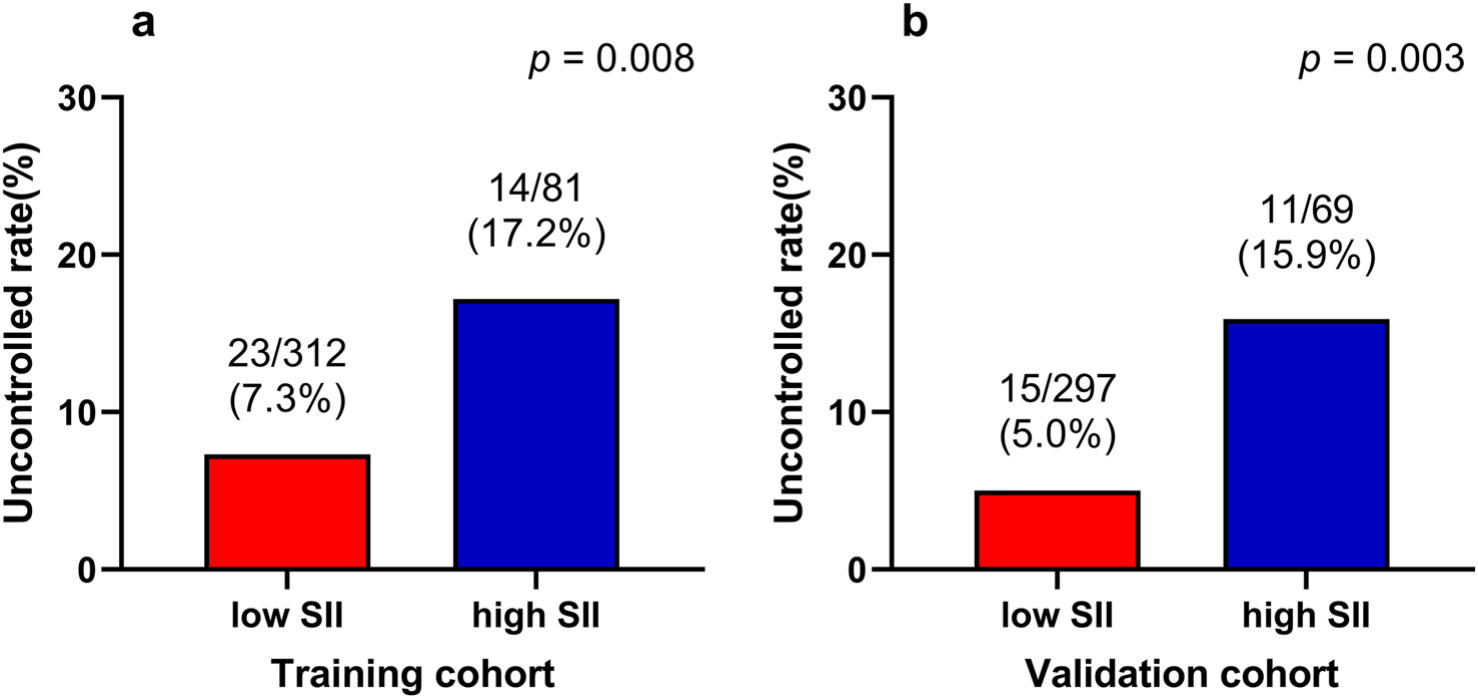
Uncontrolled rates between different groups based on SII during treatment. **a** Training cohort; **b** Validation cohort

In the multivariate logistic regression analysis, only the SII during treatment was the risk factor for the uncontrolled in the training cohort and validation cohort (all *p* < 0.05) (Table 6). The graph for uncontrolled rate of SII during treatment subgroups is shown in Fig. 4.

**Table 6:** Multivariate logistic regression analysis of different characteristics as a function of uncontrolled rate (Uncontrolled, Non-uncontrolled) in training and validation cohorts

Variables	Multivariate analysis	
	OR (95% CI)	*p*
Training cohort (*n* = 393)		
AJCC stage (8th) (III–IVb vs. I–II)	3.918 (0.898–17.098)	0.069
Metastasis (Yes vs. No)	2.281 (0.665–7.819)	0.190
EBV DNA (positive vs. negative)	2.063 (0.929–4.581)	0.075
SII during treatment (> 937.32 vs. ≤ 937.32)	2.684 (1.279–5.631)	0.009^*^
Validation cohort (*n* = 366)		
Age (> 55 vs. ≤ 55)	3.495 (1.521–8.031)	0.003^*^
SII during treatment (> 937.32 vs. ≤ 937.32)	3.498 (1.503–8.142)	0.004^*^

*p < 0.05

*AJCC* American Joint Committee on Cancer, *EBV DNA* Epstein-Barr virus DNA, *SII* Systemic immune-inflammation index

### ROC curves analysis

**Fig. 5:**
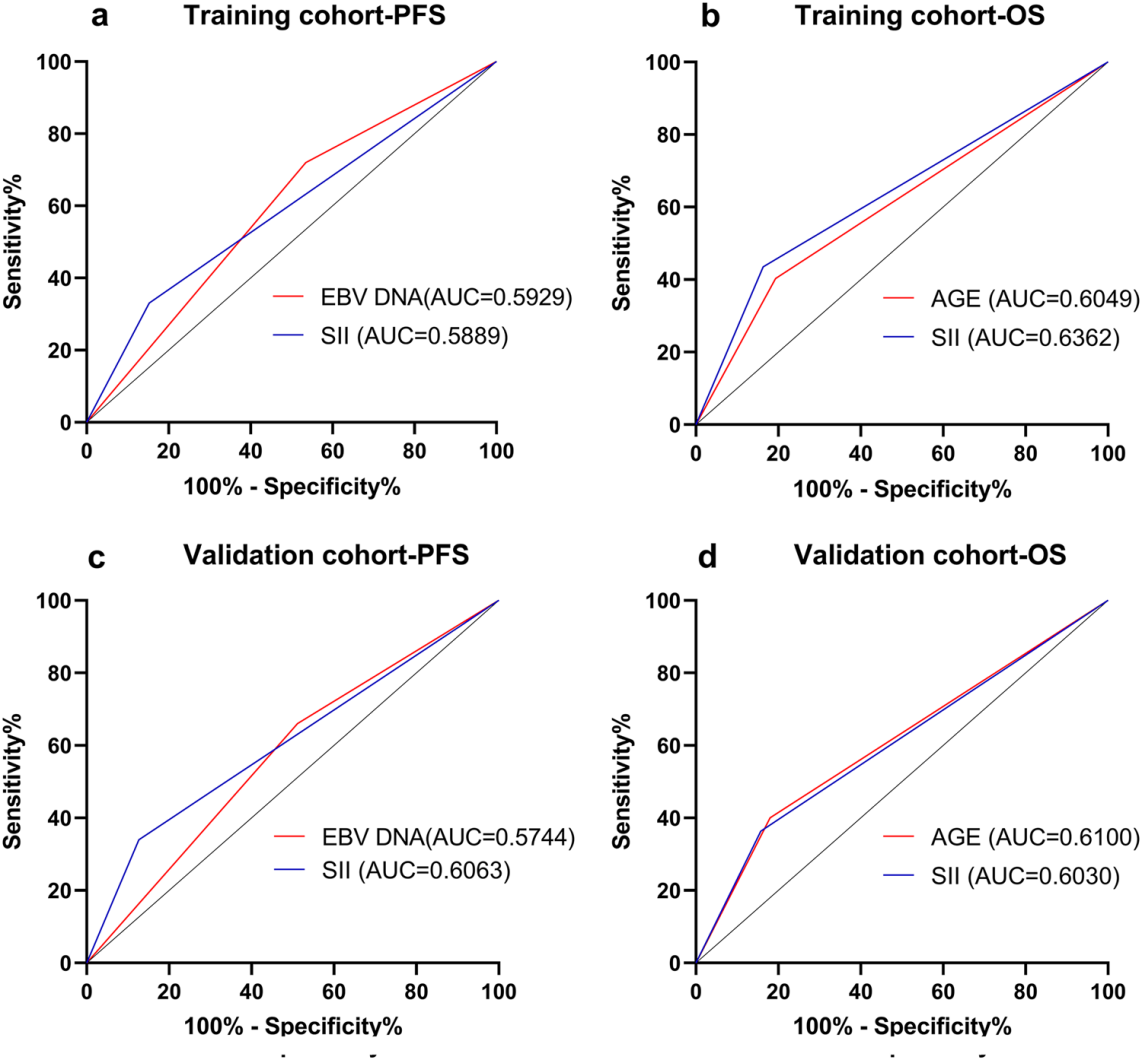
ROC curves analysis for comparing the prognostic potential of independent prognostic factors. **a** Prediction of PFS in training cohort; **b** Prediction of OS in training cohort; **c** Prediction of PFS in validation cohort; **d** Prediction of OS in validation cohort

By comparing the ROC curves, the SII during treatment demonstrated slightly smaller area under the curve (AUC) values than EBV DNA for predicting NPC progression in training cohort. However, the SII during treatment demonstrated larger area under the curve (AUC) values than EBV DNA for predicting NPC progression in validation cohort. For predicting NPC mortality, the SII during treatment demonstrated larger area under the curve (AUC) values than age in training cohort, however, the SII during treatment demonstrated slightly smaller area under the curve (AUC) values than age in validation cohort (Fig. 5).

## Discussion

In recent years, important progress in comprehensive therapies including chemotherapy, radiotherapy, targeted therapy, and immunotherapy has significantly improved survival time and quality of life for NPC patients (Lee et al. 2015; Chen et al. 2019). There are still around 10% of patients who have residual disease or develop recurrent disease which we called uncontrolled in this study, and these patients have extremely poor overall survival (Liu et al. 2019). Therefore, finding a prognosticator that can predict uncontrolled occurrence has great clinical value for NPC patients.

Inflammation is one of the seven characteristics of cancer, causing approximately 25% of new cancer cases worldwide (Schetter et al. 2010). There are increasing data showing that inflammation is closely related with tumorigenesis, proliferation, angiogenesis, metastasis, and other processes (Mantovani et al. 2008; Hanahan and Weinberg 2011). The mechanism by which high inflammatory parameters lead to poor prognosis in cancer patients remains controversial. The prognostic value of the inflammatory parameters can be explained by the role of its components. Circulating neutrophils secreted large arginase, nitric oxide, and ROS which can cause T cell activation disorder (Kusumanto et al. 2003; Müller et al. 2009; Moses and Brandau 2016). Lymphocytes play critical roles in the host immune response through inhibiting the proliferation and metastasis of cancer cells (Marra et al. 2014). Platelets in tumor patients can protect circulating tumor cells (CTCs) from shear stresses in the circulation, induce CTCs epithelial-mesenchymal transition (EMT), and promote the penetration of CTCs to metastatic sites (Labelle et al. 2011; Gil-Bernabe et al. 2012; Placke et al. 2012). NPC is typically characterized by the infiltration of inflammatory cells, which has an important role in inflammation. Accordingly, the cooperation between these inflammatory cells in the microenvironment of tumor inflammation may contribute to the tumorigenesis and cancer progression.

In this study, we found that high SII during treatment were closely related to worse 5-year PFS and OS in NPC patients, and this result was verified by the validation cohort. As a new and widely used prognostic indicator, previous studies have confirmed that pre-treatment SII have independent prognostic value in several solid tumors including NPC (Hu et al. 2014; Jiang et al. 2017; De Giorgi et al. 2019; Ji and Wang 2020). But to the best of our knowledge, the performance of the SII during treatment for predicting prognosis in NPC patients has not been investigated. In addition, we considered that the changes of inflammation/immune status during treatment should not be ignored, because it can help judge the sensitivity of the patient’s treatment. These findings confirmed that the SII during treatment could also have independent prognostic value like the pre-treatment SII in patients with NPC. And this is consistent with the poor prognosis of patients with high inflammation, high coagulation, and low lymphocytes as currently considered by scholars (Kusumanto et al. 2003; Marra et al. 2014; Moses and Brandau 2016). Regretfully, whether the prognostic value of SII during treatment was greater than that of EBV DNA and age was not confirmed in the validation cohort. This result needs to be further explored in future studies.

Another important finding from our study was that the high SII during treatment group had higher uncontrolled rate than the low SII during treatment group. Although statistical analysis of EBV DNA in the training cohort also yielded the same result, after verification in the validation cohort, only the SII during treatment still remained the statistical difference. Uncontrolled was an easily overlooked link in clinical practice and is of great significance to the integral prognosis of patients. Nonlocal failure, which the definition is similar to uncontrolled had been confirmed to be related to immune-inflammatory response in non-small-cell lung cancer (Cannon et al. 2015). It was reported that the nonlocal failure rate of patients in the high PLR (platelet-to-lymphocyte ratio) group was significantly higher than that of the patients in the low PLR group. In previous studies, the use of anti-inflammatory drugs can benefit cancer patients with high SII status (Kim et al. 2013; van Staalduinen et al. 2016). Our study found that the higher the SII during treatment, the higher the uncontrolled rate. Combining the above content, we considered that the SII during the treatment could give us predictions about the prognosis and treatment failure, and help us better evaluate the treatment effect. At present, the TNM staging of nasopharyngeal carcinoma can only reflect the size and progression of the tumor from an anatomical point of view, and cannot identify high-risk patients more effectively (Lee et al. 2012; Pan et al. 2016). Peripheral blood cells must always be monitored during treatment to evaluate the side effects of the patient's treatment. According to our study, we found changes in SII during treatment are closely related to prognosis and uncontrolled rate of NPC patients. Considering the above results, we hope that by monitoring the SII during the treatment combined with the risk stratification of patients in the same stage will help in the decision for high-risk patients to further improve the radiotherapy and chemotherapy regimens, or increase targeted therapy or even further immunotherapy. This study can assist TNM staging without increasing the burden on patients and treatment risks, and assist clinicians in obtaining better clinical decision-making, and formulate personalized treatment for patients by using the results of peripheral blood cells monitoring during treatment.

Our study had made some improvements compared with previous studies. To the best of our knowledge, we were the first to study the prognostic value of SII in NPC patients during treatment. In addition, we explored the possible risk factors for uncontrolled occurrence of NPC patients. However, our study has some limitations, this study is a retrospective study, and there may be potential deviations. Therefore, the follow-up still needs to conduct more large-scale prospective, multicenter, randomized clinical trials for further verification and research.

## Conclusion

In summary, our study demonstrated that SII during treatment was an independent prognostic predictor for PFS and OS in NPC patients, and could provide a prognostic and risk value for uncontrolled occurrence of NPC patients. Therefore, through monitoring SII during treatment, we can better evaluate the treatment effect and formulate personalized treatment.

## Supplementary Information

Below is the link to the electronic supplementary material.

Supplementary file1 (DOC 15 KB)

Supplementary file2 (DOC 13 KB)

Supplementary file3 (DOC 48 KB)

## Data Availability

The data used to support the findings of this study are available from the corresponding author upon request.
