# Supplementary material for: Systemic immune-inflammation index during treatment predicts prognosis and guides clinical treatment in patients with nasopharyngeal carcinoma
Source: J Cancer Res Clin Oncol. 2023 Jan 3;149(1):191–202. doi: 10.1007/s00432-022-04506-z (PMC9889477; doi:10.1007/s00432-022-04506-z)
Supplement: Supplementary file 1 — Supplementary file1 (DOC 15 KB) [file 432_2022_4506_MOESM1_ESM.doc]

**Supplementary file 1** Treatment method

All patients developed treatment plans according to the latest NCCN (National Comprehensive Cancer Network) guidelines at that time. All patients received Intensity-modulated radiation therapy (IMRT), patients in stage Ⅰ received IMRT alone, patients in stage Ⅱ received concurrent chemoradiotherapy, patients in stage Ⅲ/Ⅳ received concurrent chemoradiotherapy, induction chemotherapy, and/or adjuvant chemotherapy.

The principles of chemotherapy were as follows: All chemotherapy regimens were administered every 3 weeks as a complete cycle. Induction chemotherapy (IC) (1-2 cycles) and adjuvant chemotherapy (AC) (1-4 cycles) included TP, TPF, and PF. The TP regimen was paclitaxel 135 mg/m2/d or docetaxel 60 mg/m2/d on day 1 and cisplatin 25 mg/m2/d on days 1 to 3, the TPF regimen was paclitaxel 135 mg/m2/d or docetaxel 60 mg/m2/d on day 1, cisplatin 25 mg/m2/d on days 1 to 3, and 5-fluorouracil 600 mg/m2/d on days 1 to 5, and the PF regimen was cisplatin 25 mg/m2/d on days 1 to 3 and 5-fluorouracil 600 mg/m2/d on days 1 to 5. The concurrent chemoradiotherapy (CCRT) (2 cycles) included cisplatin monotherapy and TP. The cisplatin monotherapy regimen was cisplatin 25 mg/m2/d on days 1 to 3, and the TP regimen was paclitaxel 135 mg/m2/d or docetaxel 60 mg/m2/d on day 1 and cisplatin 25 mg/m2/d on days 1 to 3.

The principles of radiation therapy were as follows: all patients received 2.12-2.24 Gy radiation dose each time, 5 times a week, 6-8 weeks in total. The total prescribed IMRT doses were 70-74 Gy to the gross tumor volume of the nasopharynx (GTVnx), 66-70 Gy to the positive neck lymph node area (GTVnd), 60-62 Gy to the high-risk sites defined as clinical target volume (CTV1), and 50-56 Gy to the low-risk sites defined as clinical target volume (CTV2).
