# Supplementary material for: Systemic immune-inflammation index during treatment predicts prognosis and guides clinical treatment in patients with nasopharyngeal carcinoma
Source: J Cancer Res Clin Oncol. 2023 Jan 3;149(1):191–202. doi: 10.1007/s00432-022-04506-z (PMC9889477; doi:10.1007/s00432-022-04506-z)
Supplement: Supplementary file 2 — Supplementary file2 (DOC 13 KB) [file 432_2022_4506_MOESM2_ESM.doc]

**Supplementary file 2** The detection method for EBV-DNA

Plasma EBV DNA was measured in the Laboratory Medicine Center of Nanfang Hospital, Southern Medical University. Venous blood samples (5 ml/each case) were collected before treatment and put in ethylenediaminetetraacetic acid (EDTA) tubes. The collected blood was then centrifuged at 1500g for 5 min at 4°C. Plasma total DNA was extracted using the QIAamp blood kit (Qiagen, Hilden, Germany). The EBV genome was amplified by real-time quantitative PCR (RT-qPCR) using an EBV RT-qPCR kit and primers 5′-GCTGCGCTGCTGCTATCTT-3′ (forward) and 5′-CAAGCCCACTCCCCTGTCT-3′ (reverse) according to the manufacturer’s instructions (Liferiver, Shanghai, China). The GAPDH gene was amplified as a control using the primers 5′-GGCGACGCAAAAGAAGATG-3′ (forward) and 5′-CCGTTGACTCCGACCTTCAC-3′ (reverse). PCR conditions were as follows: initial denaturation at 95°C for 10 minutes, followed by 40 cycles of denaturation at 95°C for 15 seconds and amplification at 56°C for 1 minute.
