# Supplementary material for: Systemic immune-inflammation index during treatment predicts prognosis and guides clinical treatment in patients with nasopharyngeal carcinoma
Source: J Cancer Res Clin Oncol. 2023 Jan 3;149(1):191–202. doi: 10.1007/s00432-022-04506-z (PMC9889477; doi:10.1007/s00432-022-04506-z)
Supplement: Supplementary file 3 — Supplementary file3 (DOC 48 KB) [file 432_2022_4506_MOESM3_ESM.doc]

| **Supplementary Table 1.** Baseline characteristics of the Training cohort (393) and Validation cohort (366) | | | |
| --- | --- | --- | --- |
| Variables | Training cohort  No. (%) | Validation cohort  No. (%) | *p* |
| Gender |  |  | 0.393 |
| male | 288 (73.3) | 258 (70.5) |  |
| female | 105 (26.7) | 108 (29.5) |  |
| Age |  |  | 0.657 |
| ≤ 55 | 304 (77.4) | 288 (78.7) |  |
| > 55 | 89 (22.6) | 78 (21.3) |  |
| EBV DNA status |  |  | 0.321 |
| negative | 161 (41.0) | 163 (44.5) |  |
| positive | 232 (59.0) | 203 (55.5) |  |
| AJCC stage (8th) |  |  | 0.567 |
| Ⅰ-Ⅱ | 73 (18.6) | 74 (20.2) |  |
| Ⅲ-Ⅳb | 320 (81.4) | 292 (79.8) |  |
| Tumor stage |  |  | 0.787 |
| T1-T2 | 153 (38.9) | 146 (39.9) |  |
| T3-T4 | 240 (61.1) | 220 (60.1) |  |
| Node stage |  |  | 0.548 |
| N0-N1 | 168 (42.7) | 149 (40.6) |  |
| N2-N3 | 225 (57.3) | 218 (59.4) |  |
| Metastasis |  |  | 0.699 |
| Non-metastasis | 377 (95.9) | 349 (95.4) |  |
| Metastasis | 16 (4.1) | 17 (4.6) |  |
| WHO pathologic type |  |  | 0.297 |
| TypeⅠ | 1 (0.2) | 3 (0.8) |  |
| TypeⅡ | 26 (6.6) | 32 (8.7) |  |
| TypeⅢ | 366 (93.2) | 331 (90.5) |  |
| SII |  |  | 0.543 |
| ≤ 937.32 | 312 (79.4) | 297 (81.1) |  |
| > 937.32 | 81 (20.6) | 69 (18.9) |  |
| PLR |  |  | 0.579 |
| ≤ 311.86 | 213 (54.2) | 191 (52.2) |  |
| > 311.86 | 180 (45.8) | 175 (47.8) |  |
| SII: Systemic immune-inflammation index; PLR: Platelet-lymphocyte ratio; EBV DNA: Epstein-Barr virus DNA; AJCC: American Joint Committee on Cancer; WHO: World Health Organization | | | |
